# Numerical Study of Concentration Polarization in Electrodialysis for High-Salinity Solution Concentration in Air-Conditioning Systems

**DOI:** 10.3390/membranes16080259

**Published:** 2026-07-29

**Authors:** Bo Sun, Ning Lyu

**Affiliations:** School of Energy and Power, Jiangsu University of Science and Technology, Zhenjiang 212003, China; lvning@just.edu.cn

**Keywords:** electrodialysis, concentration polarization, ion-exchange membrane, liquid desiccant, high-salinity solution

## Abstract

Concentration polarization is a common phenomenon in membrane separation processes and generally impairs mass transfer efficiency. Electrodialysis (ED) is considered a promising technology for concentrating high-salinity solutions used in air-conditioning systems; however, concentration polarization under high-concentration operating conditions remains insufficiently understood. In this study, a numerical framework combining a simplified model and a coupled transport model was developed to characterize concentration distributions within an ED concentrate channel. The effects of flow velocity, current density, and feed concentration on concentration profiles were systematically investigated. The results show that transmembrane water transport plays an important role in concentration polarization, and neglecting this effect leads to significant overestimation of ion concentration near the membrane surface. Although ion concentration increases markedly in the vicinity of the ion-exchange membranes, it remains nearly constant in the bulk region along the flow direction. Based on this non-uniform concentration distribution, a conceptual ED configuration with separated flow channels was proposed and evaluated. The results indicate that selectively extracting the enriched boundary-layer region can enhance the outlet concentration of the product stream, whereas increasing the intermediate channel width reduces volumetric yield, revealing a clear trade-off between concentration enhancement and production capacity.

## 1. Introduction

High-salinity solutions, such as aqueous lithium chloride (LiCl) solutions with salt concentrations of approximately 30 wt.%, are widely employed in various air-conditioning systems, including liquid desiccant dehumidification systems [1,2] and heat-source tower heat pump systems [3], to enhance dehumidification and heating performance. Owing to their hygroscopic nature, the concentration of these electrolyte solutions gradually decreases during operation. Consequently, reconcentration is essential for restoring moisture absorption capacity and maintaining a sufficiently low freezing point [4,5]. In air-conditioning applications, this reconcentration process is generally referred to as solution regeneration [6,7,8]. Beyond air-conditioning systems, the concentration of high-salinity streams, such as industrial effluents containing heavy metals, saline wastewater, and process streams containing valuable salts, is also required for environmental protection and sustainable industrial production [9,10,11,12]. Therefore, efficient concentration of high-salinity solutions is of considerable importance across multiple industrial sectors.

Thermal evaporation technologies, including membrane distillation (e.g., Thermo-osmotic membrane distillation-crystallization) [13], mechanical vapor recompression [14], and multiple-effect evaporation [15], are widely used for concentrating high-salinity solutions. However, their relatively high energy consumption and substantial capital investment often limit their economic feasibility, particularly for small-scale concentration processes [16]. Reverse osmosis (RO), which employs semi-permeable membranes to separate water from saline solutions, is highly sensitive to salinity and is therefore unsuitable for treating high-salinity streams [17]. Electrodialysis (ED) is a membrane-based separation process that utilizes ion-exchange membranes and an externally applied electric field to either desalt or concentrate electrolyte solutions [18,19,20]. Previous studies have reported that ED can treat solutions with salinities exceeding 100 g/L while maintaining energy consumption within the range of 7–15 kWh/m^3^ [21]. Furthermore, several investigations have suggested that ED may serve as a potential alternative to conventional thermal evaporation technologies for concentrating high-salinity electrolyte solutions under suitable operating conditions [22,23,24,25]. Consequently, ED is regarded as an energy-efficient and promising technology for concentrating high-salinity streams.

Transport phenomena in ED involve the coupled migration of ions and water through ion-exchange membranes and along flow channels under concentration and electric potential gradients. When an electric field is applied across an ED stack, anions migrate through anion-exchange membranes (AEMs), while cations migrate through cation-exchange membranes (CEMs). As a result, ions accumulate near the membrane/solution interface in the concentrate compartment and deplete near the corresponding interface in the dilute compartment, leading to concentration variations along the membrane surfaces. Owing to finite ion diffusivity, concentration gradients develop between the membrane surface and the bulk solution under steady-state conditions. In the concentrate compartment, excessive ion accumulation near the membrane surface may induce salt precipitation if the local concentration exceeds the solubility limit. In the dilute compartment, ion depletion at the membrane surface may lead to limiting-current conditions [26,27]. Such concentration gradients near membrane/solution interfaces are generally referred to as concentration polarization, a phenomenon commonly observed in membrane-based separation processes, including pervaporation, ED, membrane distillation, and reverse osmosis [28,29,30,31]. In the present study, the term “concentration polarization” refers broadly to the formation of concentration gradients near interfaces rather than specifically to the ion-depletion effect associated with limiting-current behavior in dilute channels.

Concentration polarization inevitably affects mass-transfer efficiency and increases the energy consumption of membrane separation processes. Therefore, understanding concentration polarization in ED is essential for improving the performance of ED systems in various applications. Numerous studies have investigated concentration polarization in ED for desalination, water treatment, and other relatively low-salinity applications. Mitko et al. [32] estimated concentration distributions along ED channels using a simplified linear concentration-gradient model. Law et al. [33] adopted a nonlinear concentration-distribution model to predict concentration profiles and boundary-layer thicknesses more accurately. Mas et al. [34] examined salt distribution in ED compartments while considering factors such as potential drop, electrolyte flow velocity, and membrane spacing. Tedesco et al. [35] developed a two-dimensional model incorporating non-ideal membrane behavior and solved the Nernst–Planck equation in both membranes and flow channels. Zourmand et al. [36] established a computational fluid dynamics (CFD)-based mass-transfer model to predict concentration distributions and ion transport by simultaneously solving the Navier–Stokes and steady-state Nernst–Planck equations.

Although extensive efforts have been devoted to concentration polarization in ED desalination and low-salinity applications, relatively little attention has been paid to ED processes involving high-salinity solutions. Under high-concentration conditions, ion transport properties, water transport behavior, and membrane characteristics can differ substantially from those under dilute conditions. Strong osmotic water transport, concentration-dependent membrane properties, and nonlinear concentration polarization effects make it considerably more challenging to accurately predict concentration distributions in ED channels operating at high salinity.

In this study, concentration polarization in ED for concentrating high-salinity solutions is numerically investigated. Two modeling approaches are employed: a simplified model considering only salt transport across the membranes and a coupled model accounting for the coupled transport of salt and water. Although the governing transport equations are adapted from established ED modeling frameworks, the present work focuses on their application to high-salinity LiCl concentration, where concentration-dependent salt/water transport and boundary-layer enrichment become particularly important. The effects of key operating parameters, including flow velocity, current density, and inlet salt concentration, on concentration distributions within the concentrate channel are systematically analyzed. In addition, a conceptual ED concentration process utilizing separated flow channels is proposed to explore the potential utilization of concentration polarization for enhanced concentration performance. The developed model is intended to provide a useful framework for predicting concentration distributions under a wide range of operating conditions and to contribute to the advancement of electrically driven separation technologies for high-salinity solution concentration.

## 2. Mathematical Modeling

### 2.1. Model Description

**Figure 1:**
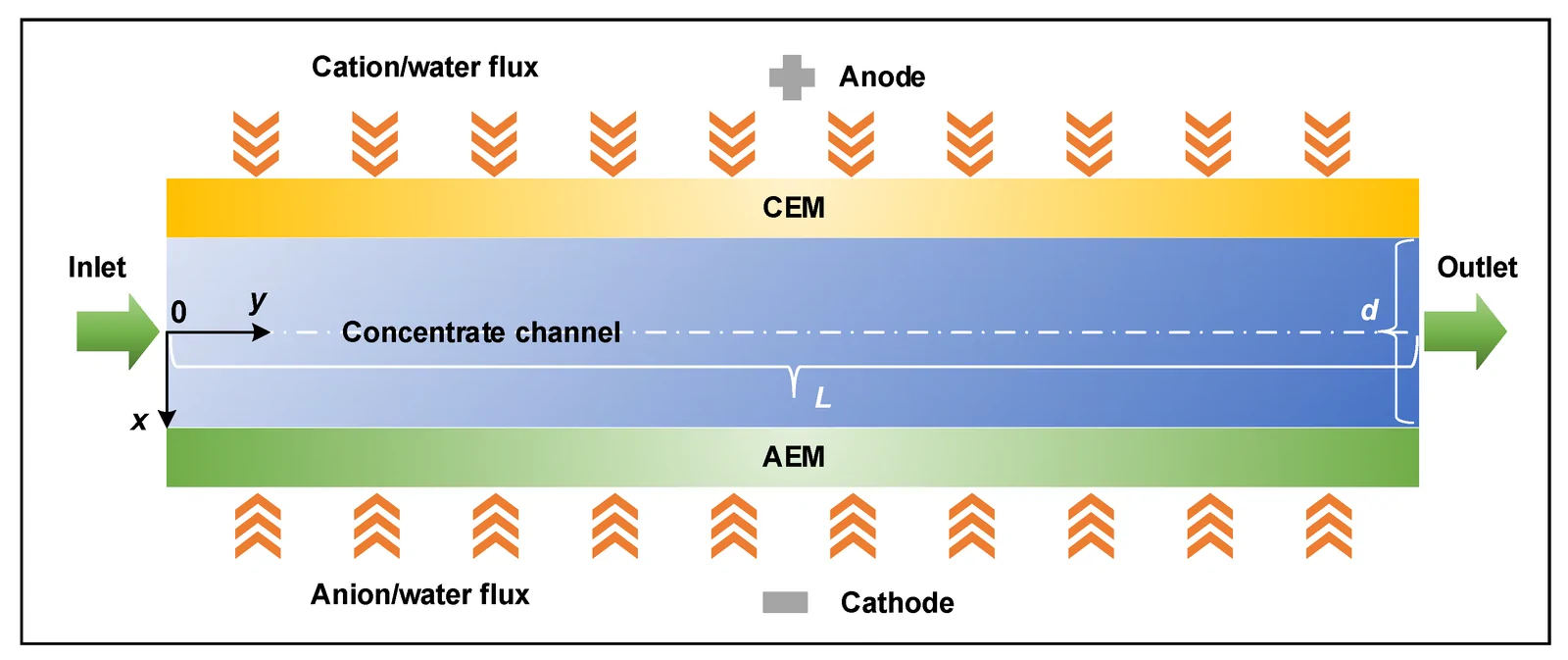
Schematic diagram of the geometric model. The key dimensions of the channel are its length, denoted as *L*, and its width (the spacing between membranes), denoted as *d*.

In the present study, the modeling focuses on a single representative concentrate channel within an ED stack to calculate the corresponding concentration and velocity distributions. A schematic illustration of the computational domain is presented in Figure 1. The domain consists of a cation-exchange membrane (CEM), an anion-exchange membrane (AEM), and the concentrate channel enclosed between them. The channel length and width are 160 mm and 8 mm, respectively. The channel width was set to 8 mm according to the laboratory-scale ED configuration used for high-salinity LiCl regeneration; this geometry was used as the basis for the present channel-scale numerical analysis. Because the fluid velocity considered in this study is relatively low (≤0.1 m/s), the flow inside the channel is assumed to remain laminar (the Reynolds number for all simulated cases is below 200). As the electrolyte solution flows through the channel, coupled ion and water transport across the ion-exchange membranes from adjacent dilute channels results in a gradual increase in outlet concentration.

The ED process is modeled under constant-current operation, which is particularly relevant for high-salinity applications. Rather than explicitly resolving the detailed internal transport processes within the ion-exchange membranes, the effects of transmembrane transport are incorporated through flux boundary conditions imposed at the membrane/channel interfaces. These boundary conditions account for the net transport of salt and water arising from electro-migration and osmosis. Such a treatment substantially simplifies the numerical model while preserving the essential physics governing concentration polarization within the concentrate channel.

According to our previous studies [16,37], the salt and water fluxes through a membrane pair can be expressed as follows:

(1) $$J_{s}=\frac{t_{s}}{F}i-\alpha (C_{c}-C_{d})\;v_{w}=\frac{n_{h}t_{s}V_{m,w}}{F}i+\beta (C_{c}-C_{d})$$

where $J_{s}$ is the salt flux, $v_{w}$ is the water flux, $t_{s}$ is the salt transport number, F is Faraday constant, i is the current density, α is the overall salt permeability, β is the overall osmosis coefficient, $n_{h}$ is the effective salt hydration number, $V_{m,w}$ is the molar volume of water, and $C_{c}$ and $C_{d}$ denote the salt concentrations in the concentrate and dilute compartments, respectively. The concentration dependence of the overall salt permeability and osmotic coefficient has been discussed in detail in our previous work [37]. In this study, the ion-exchange membranes were treated as effective flux boundaries, and the membrane-related parameters in Equation (1) were evaluated using bulk-concentration-based empirical correlations. Since the concentration difference between the dilute and concentrate compartments is much larger than the near-membrane concentration deviation within the concentrate channel, this simplification is expected to have a limited influence on the calculated fluxes.

An aqueous lithium chloride (LiCl) solution is employed as the working fluid in this study. Relevant thermophysical properties, including density and dynamic viscosity, are obtained from the correlations proposed by Conde [38]. The diffusion coefficient of LiCl exhibits only slight variation over a broad concentration range, remaining approximately within 1.3 × 10^−9^ to 1.5 × 10^−9^ m^2^/s for concentrations between 0 and 5 mol/L [39]. It should be noted that the present study focuses on LiCl at a fixed operating temperature because LiCl is a representative liquid desiccant for air-conditioning applications. Similar concentration-polarization behavior is expected for other electrolytes in ED concentration processes, although the quantitative results may vary with electrolyte-specific properties such as density, viscosity, diffusivity, osmotic behavior, and membrane transport parameters.

The salt transport number, $t_{s}$, and effective salt hydration number, $n_{h}$, were experimentally determined in our previous study from the measured salt and water transport during ED concentration experiments [37]. Among the influencing factors investigated, salt concentration was found to exert the most significant influence on these parameters. Accordingly, empirical correlations for LiCl solution were established as follows [16,37]:

(2) $$t_{s}=0.93-6.84\times 10^{-5}\cdot C\;n_{h}=13.78\cdot exp(-\frac{C}{3697})+3.27$$

where C represents the molar concentration of the bulk LiCl solution (mol/m^3^).

To facilitate model development, several assumptions are adopted in this study, consistent with commonly used approaches in the literature [35]: (1) The process operates at steady state, and the flow regime is laminar. (2) The velocity field and the concentration field are solved independently. (3) The fluid velocity profile is assumed to be fully developed at the channel inlet. (4) The electrolyte (LiCl) is considered fully dissociated. The anion (Cl^−^) and cation (Li^+^) are assigned equal diffusion coefficients, denoted by $D_{-}=D_{+}=D_{s}$. (5) The anion-exchange membrane (AEM) and cation-exchange membrane (CEM) are assumed to possess identical relevant properties, including thickness, ion-exchange capacity, ion diffusivity, and perm-selectivity. (6) The osmotic and electro-osmotic water fluxes across the AEM and CEM are considered equal. Therefore, the total water flux across the anion- and cation-exchange membranes is the same, with $v_{w,AEM}=v_{w,CEM}=1/2v_{w}$. (7) The ED process is assumed to be isothermal, with all properties evaluated at a constant operating temperature.

These assumptions enable a tractable numerical formulation while retaining the essential transport mechanisms governing concentration polarization in high-salinity ED systems. The assumptions of identical AEM/CEM properties and equal water fluxes were adopted to establish a simplified symmetric baseline model. In practical ED systems, AEMs and CEMs may differ in perm-selectivity, ion-exchange capacity, ion diffusivity, co-ion transport, water uptake, and electro-osmotic water transport, which could lead to asymmetric concentration boundary layers and affect the predicted outlet concentration. Therefore, the present results should be interpreted as a first-order analysis of the main concentration-polarization mechanism, and future work should incorporate membrane-specific parameters and sensitivity analysis to assess model robustness.

### 2.2. Simplified Model

A simplified model was first developed in which transmembrane water transport was neglected and only ion transport across the ion-exchange membranes was considered. Under this assumption, the velocity distribution inside the concentrate channel can be approximated as a fully developed parabolic flow profile. Furthermore, because the channel length is substantially greater than the channel width, axial diffusion in the flow direction is assumed negligible compared with transverse diffusion. This assumption is commonly adopted in ED channel modeling and significantly simplifies the governing transport equations.

Accordingly, the steady-state concentration distribution within the concentrate channel can be described using the following two-dimensional convection–diffusion equation [35]:

(3) $$D\frac{\partial^{2}C(x,y)}{\partial x^{2}}=u\left(x\right)\frac{\partial C\left(x,y\right)}{\partial y},\;-\frac{d}{2}\leq x\leq \frac{d}{2},\;0\leq y\leq L$$

where D is the ion diffusion coefficient and $u\left(x\right)$ is the local axial velocity. Because the flow is assumed to be fully developed and laminar, the velocity profile follows the classical Hagen–Poiseuille equation [35]:

(4) $$u(x)=\frac{3}{2}u_{\mathrm{ave}}\left(1-4{\left(\frac{x}{d}\right)}^{2}\right),\;-\frac{d}{2}\leq x\leq d/2$$

where $u_{\mathrm{ave}}$ denotes the average flow velocity within the channel.

The governing convection–diffusion equation is solved subject to the following boundary conditions:

(5) $$D\frac{dC}{dx}=\pm J_{s},\;x=\mp \frac{d}{2}\;C(x,0)=C_{0}$$

where $C_{0}$ is the inlet concentration of the concentrate channel.

The governing equation was discretized using the forward finite-difference method. Employing a structured uniform computational grid, the discretized form of Equation (3) can be expressed as follows:

(6) $$C_{i+1,j}=R_{i,j}(C_{i,j+1}+C_{i,j-1})+(1-2R_{i,j})C_{i,j}$$

where $R_{i,j}$ is the local step ratio defined by:

(7) $$R_{i,j}=\frac{D∆y}{u_{i,j}(∆x{)}^{2}}$$

where ∆x and ∆y represent the grid spacing in the transverse and axial directions, respectively.

The simplified model was implemented in MATLAB (version 2016b) because it is based on a reduced convection-diffusion equation with a prescribed fully developed velocity profile and was mainly used for rapid comparison with the coupled model. The computational domain representing the concentrate channel was discretized into *m* rows and *n* columns, corresponding to grid spacings of ∆*y* = *L*/*m* and ∆*x* = *d*/*n*.

To ensure numerical accuracy and eliminate potential grid dependency, a mesh-independence analysis was conducted. Several mesh densities were tested, and the predicted concentration distributions were compared. A mesh consisting of approximately 250,000 computational cells (*m* × *n* = 5000 × 50) was ultimately adopted because further mesh refinement produced negligible changes in the simulation results.

Although the simplified model neglects transmembrane water transport and therefore cannot fully capture the coupled transport behavior occurring near the membrane surfaces, it provides a computationally efficient framework for rapidly estimating concentration distributions and identifying the dominant transport characteristics within the ED channel.

### 2.3. Coupled Model

In practical ED systems, electro-osmotic water transport is an inherent process, and osmotic water transport becomes particularly significant under high-salinity operating conditions. To achieve a more accurate prediction of concentration distributions within the concentrate channel, transmembrane water transport must therefore be incorporated into the model. Consequently, the assumption of a purely parabolic velocity profile employed in the simplified model is no longer valid, and the velocity field must be solved simultaneously with the concentration field.

In the coupled model, the steady-state flow field of the incompressible electrolyte solution is governed by the two-dimensional Navier–Stokes equations [40]:

(8) $$\frac{\partial u}{\partial x}+\frac{\partial v}{\partial y}=0\;\rho (u\frac{\partial u}{\partial x}+v\frac{\partial u}{\partial y})=-\frac{\partial p}{\partial x}+\mu (\frac{\partial^{2}u}{\partial x^{2}}+\frac{\partial^{2}u}{\partial y^{2}})\;\rho (u\frac{\partial v}{\partial x}+v\frac{\partial v}{\partial y})=-\frac{\partial p}{\partial y}+\mu (\frac{\partial^{2}v}{\partial x^{2}}+\frac{\partial^{2}v}{\partial y^{2}})$$

where u and v denote the velocity components in the transverse (*x*) and axial (*y*) directions, respectively, ρ is the fluid density, p is the pressure, and μ is the dynamic viscosity of the fluid.

The governing equations for the velocity field are solved subject to the following boundary conditions:

(9) $$u=u_{0},\;y=0\;p=0,\;y=L\;v=\pm \frac{v_{w}}{2},\;x=\mp d/2$$

where $u_{0}$ is the inlet velocity and $v_{w}$ is the total water flux through the ion-exchange membranes.

After obtaining the velocity field, the concentration distribution within the channel is determined by solving the two-dimensional convection–diffusion equation. Unlike the simplified model, diffusion in both the transverse and axial directions is considered in the coupled model. The governing equation for the steady-state concentration field is expressed as follows:

(10) $$D(\frac{\partial^{2}C}{\partial x^{2}}+\frac{\partial^{2}C}{\partial y^{2}})=u\frac{\partial C}{\partial x}+v\frac{\partial C}{\partial y}$$

The corresponding boundary conditions are given by:

(11) $$C=C_{0},y=0\;D\frac{dC}{dx}+v_{w}C=\pm J_{s},\;x=\mp d/2$$

Compared with the simplified model, the additional convective term associated with water transport enables the coupled model to more accurately capture concentration evolution near the membrane surfaces. This improvement is particularly important under high-salinity conditions, where osmotic water transport substantially affects local concentration distributions and boundary-layer development.

**Figure 2:**
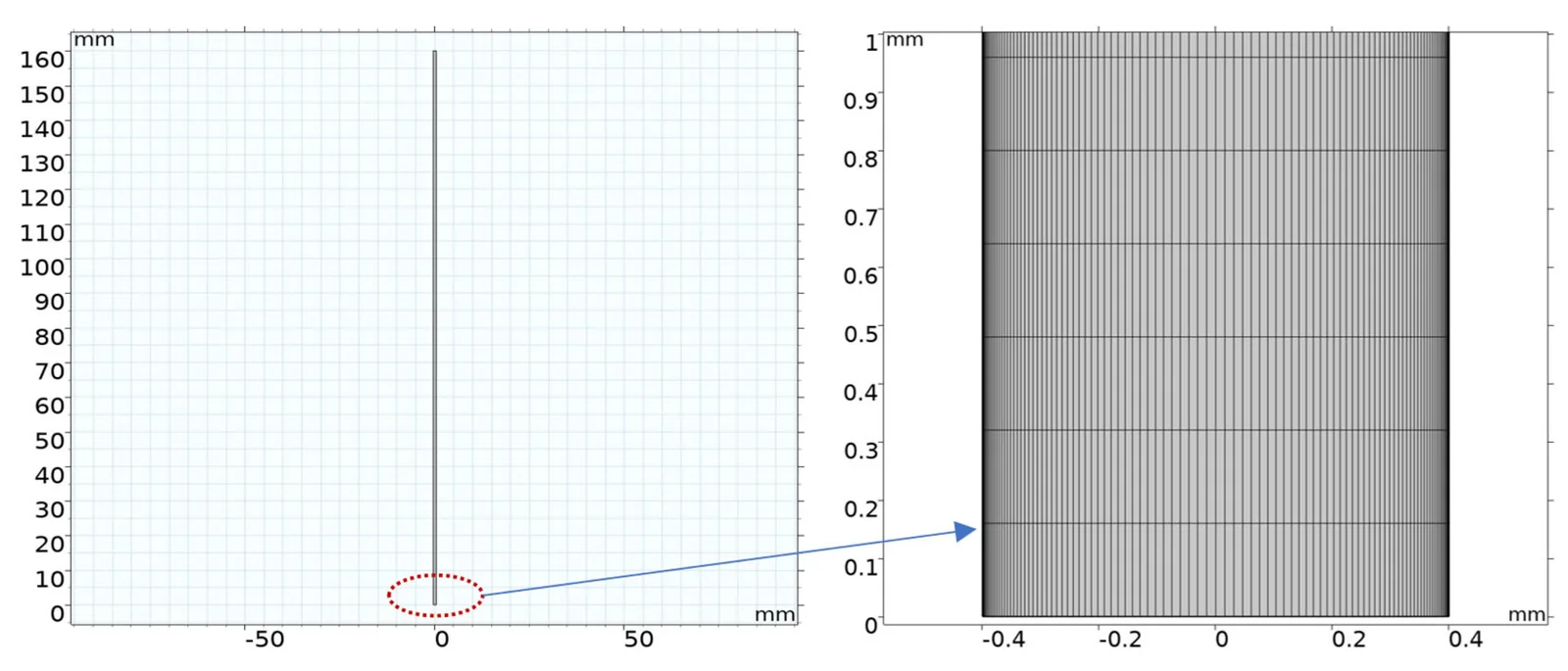
Computational mesh used in the coupled model. The left panel shows the overall concentrate-channel domain, and the right panel shows a magnified view of the locally refined mesh near the membrane surface. The axis units are given in mm.

The coupled governing equations, including the Navier–Stokes equations and the convection–diffusion equation, were solved numerically using the commercial finite-element software COMSOL Multiphysics^®^ (version 5.6). In the COMSOL implementation, the ion-exchange membranes were not treated as separate computational domains. Instead, the membrane/channel interfaces were defined as flux boundaries. The salt and water fluxes given by Equation (1) were imposed on these boundaries, and the applied current density was introduced through the corresponding salt flux term. The concentration-dependent membrane parameters in Equation (2) were evaluated using the bulk LiCl concentration. As illustrated in Figure 2, to accurately resolve the steep concentration gradients that develop near the membrane surfaces, localized mesh refinement was applied within the thin concentration boundary layers adjacent to the walls. In contrast, a relatively coarser mesh was employed in the bulk-flow region to improve computational efficiency while maintaining sufficient numerical accuracy.

A comprehensive mesh-independence analysis was conducted to verify that the numerical results were independent of grid resolution. Several mesh densities were tested and compared. A final mesh containing approximately 100,000 elements (corresponding to *m* × *n* = 1000 × 100 grid points) was adopted because further refinement produced negligible changes in the predicted velocity and concentration distributions.

To characterize the bulk transport behavior within the concentrate channel, average velocity and concentration values were calculated at different axial locations. The average velocity at a given axial position is defined as [35]:

(12) $$u_{\mathrm{ave}}(y)=\frac{\int_{-d/2}^{d/2}u(x,y)dx}{d}$$

Similarly, the flow-averaged concentration at a given axial position is calculated as:

(13) $$C_{\mathrm{ave}}(y)=\frac{\int_{-d/2}^{d/2}C(x,y)u(x,y)dx}{u_{\mathrm{ave}}(y)d}$$

A generalized averaging formulation can also be applied to arbitrary channel cross-sections:

(14) $$u_{\mathrm{ave}}=\frac{\int_{a}^{b}u(x,y)dx}{b-a}\;C_{\mathrm{ave}}=\frac{\int_{a}^{b}C(x,y)u(x,y)dx}{u_{\mathrm{ave}}(b-a)}$$

where *a* and *b* represent the boundaries of the selected cross-section.

Because high-salinity solutions are typically characterized using solute mass concentration (or fraction) rather than molar concentration, the calculated molar concentrations were converted into solute mass concentrations using the following relationship:

(15) $$\xi_{s}=\frac{{C_{s}M}_{s}}{\rho_{s}}$$

where $\xi_{s}$ is the solute mass concentration (or fraction), $M_{s}$ is the molar mass of the salt, and $\rho_{s}$ is the density of the solution.

In the subsequent analysis, all concentration results are presented in terms of solute mass concentration because this representation is more commonly adopted in the design and performance evaluation of air-conditioning systems employing liquid desiccants.

### 2.4. Overall Settings and Model Validation

The anion-exchange membranes (AEMs) and cation-exchange membranes (CEMs) employed in this study share identical geometric dimensions, each with a thickness of 0.16 mm and an effective active area of 0.0112 m^2^ (0.16 m × 0.07 m). The concentrate channel is 0.16 m in length and 0.008 m in width. Detailed electrochemical and transport properties of the ion-exchange membranes are provided in our previous study [37]. In the present model, the membrane-related parameters used to calculate the salt and water fluxes include the salt transport number, effective salt hydration number, overall salt permeability, and overall osmosis coefficient.

To investigate the effects of operating conditions on concentration and velocity distributions, a range of input parameters was considered. The inlet salt concentration (expressed as solute mass fraction) varies from 10% to 30%, the applied current density ranges from 200 to 800 A/m^2^, and the inlet flow velocity is set between 1 and 10 cm/s. The specific parameter combinations used in each simulation case are provided in the corresponding sections of the results.

Based on the governing equations and boundary conditions described above, steady-state velocity and concentration fields within the channel were obtained. To evaluate model reliability, a comparative analysis between the simplified model and the coupled model was first conducted under *i* = 400 A/m^2^, *u* = 2.5 cm/s, and *ξ*_s_ = 10% (LiCl solution).

**Figure 3:**
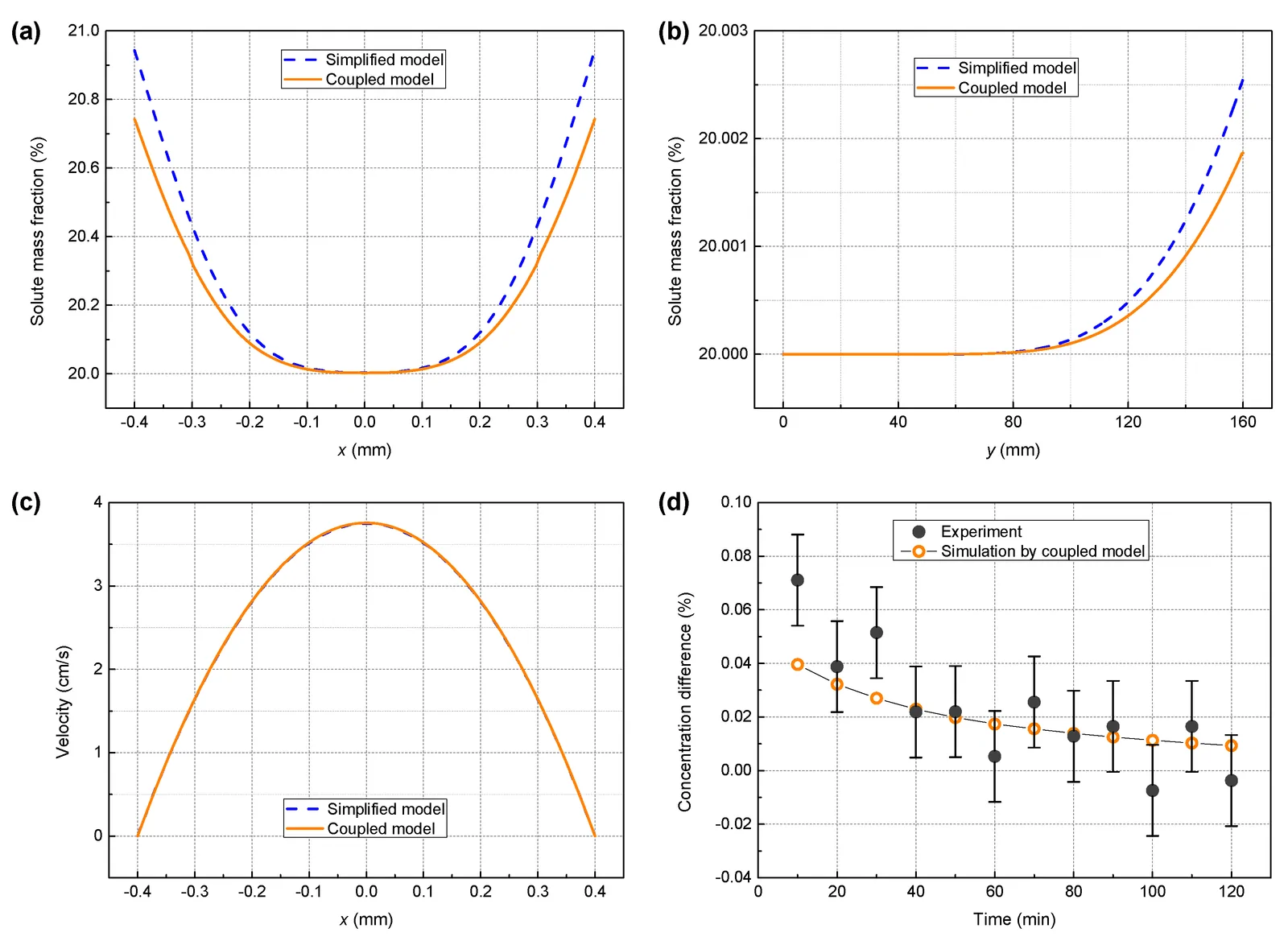
The comparison between the simplified model and the coupled model: (**a**) the cross-sectional salt concentration distribution at the outlet of the channel, (**b**) the salt concentration distribution along the center of the channel, and (**c**) the velocity distribution of solution at the outlet of the channel. (**d**) the comparison between the coupled model and experiment in terms of the concentration increase from the inlet to the outlet of the channel. The experimental data was obtained at each moment of an ED process operating in batch mode (*i* = 400 A/m^2^, *u* = 2.5 cm/s, and LiCl concentration of 10%).

Figure 3a presents the cross-sectional concentration distribution at the channel outlet (*y* = *L*). The results show that the simplified model slightly overestimates the ion concentration near the membrane surfaces. This deviation is primarily attributed to the neglect of transmembrane water transport, which otherwise contributes to dilution effects in the boundary layer. Figure 3b illustrates the concentration distribution along the channel centerline (*x* = 0). Both models show excellent agreement in the bulk region, indicating that the simplified model is capable of capturing the overall axial concentration trend under moderate operating conditions. In addition, the velocity profiles predicted by the two models at the channel outlet are nearly identical, as shown in Figure 3c, confirming the validity of the laminar fully developed flow assumption for velocity prediction.

To further validate the coupled model, the predicted concentration increase from the inlet to the outlet of the concentrate channel was compared with experimental data. The experimental setup and procedures are detailed in our previous work [37]. Experiments were conducted in batch mode at an initial LiCl concentration of 10%. This validation condition was selected because the concentration increase from the inlet to the outlet of the concentrate channel is relatively larger at 10% LiCl, providing a clearer experimental signal for model validation. At higher LiCl concentrations, the single-pass concentration increase becomes much smaller and is more susceptible to measurement uncertainty. In batch operation, the concentration difference between the concentrate and dilute streams gradually decreases over time due to the evolution of the driving force for ion transport. At each time step, experimentally measured operating parameters (including current density, flow rate, and instantaneous concentrations) were used as input to the coupled model. The corresponding outlet concentration was then computed and compared with experimental results.

Figure 3d compares the simulated and experimental concentration increase from the inlet to the outlet of the concentrate channel. The model predictions agree well with experimental data, with only minor deviations observed. These discrepancies are mainly attributed to experimental uncertainties, as the measured concentration increase is relatively small and therefore sensitive to measurement errors.

Overall, the coupled model demonstrates improved predictive accuracy compared with the simplified model, particularly in capturing concentration evolution under conditions where transmembrane water transport plays a significant role. Therefore, the coupled model is adopted in the subsequent analysis.

## 3. Results and Discussion

### 3.1. Concentration and Velocity Profiles

**Figure 4:**
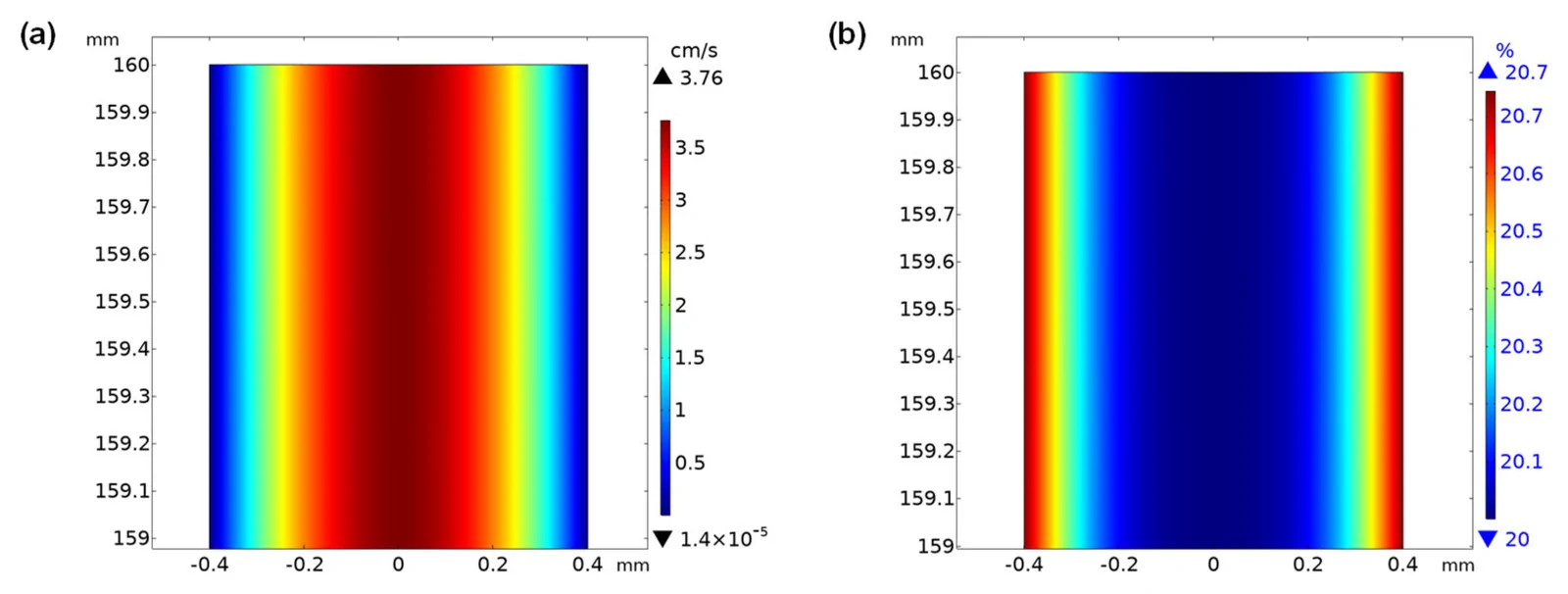
(**a**) Velocity distribution and (**b**) concentration distribution in the concentrate channel under *i* = 400 A/m^2^, *u* = 2.5 cm/s, and LiCl concentration of 20%.

Figure 4 presents the simulated velocity and concentration distributions within the concentrate channel under the operating conditions of *i* = 400 A/m^2^, *u* = 2.5 cm/s, and LiCl concentration of 20%. As shown in Figure 4a, the velocity field exhibits a typical parabolic profile characteristic of pressure-driven laminar flow in a confined channel. The velocity approaches zero at the membrane surfaces due to the no-slip boundary condition and reaches a maximum value of approximately 3.76 cm/s at the channel centerline.

In contrast, the concentration distribution shown in Figure 4b exhibits the opposite trend. The highest concentration, approximately 20.7%, occurs in the vicinity of the ion-exchange membrane surfaces. This localized enrichment is attributed to ion migration from the adjacent dilute compartments into the concentrate channel under the applied electric field. Toward the channel center, the concentration remains relatively uniform and close to the inlet bulk value, indicating that convective transport dominates in the core region while diffusion is confined primarily to a thin boundary layer adjacent to the membranes. This clearly demonstrates the formation of concentration polarization under high-salinity ED operation. The formation of this concentration profile can be understood as the result of competition between transmembrane ion migration, transverse diffusion, axial convection, and water transport. Ion migration enriches the solution near the membrane surfaces, whereas axial convection continuously transports the bulk solution downstream and maintains a relatively uniform concentration in the channel center. Therefore, the near-membrane boundary layer, rather than the entire channel cross-section, dominates the concentration-polarization behavior in the concentrate channel.

**Figure 5:**
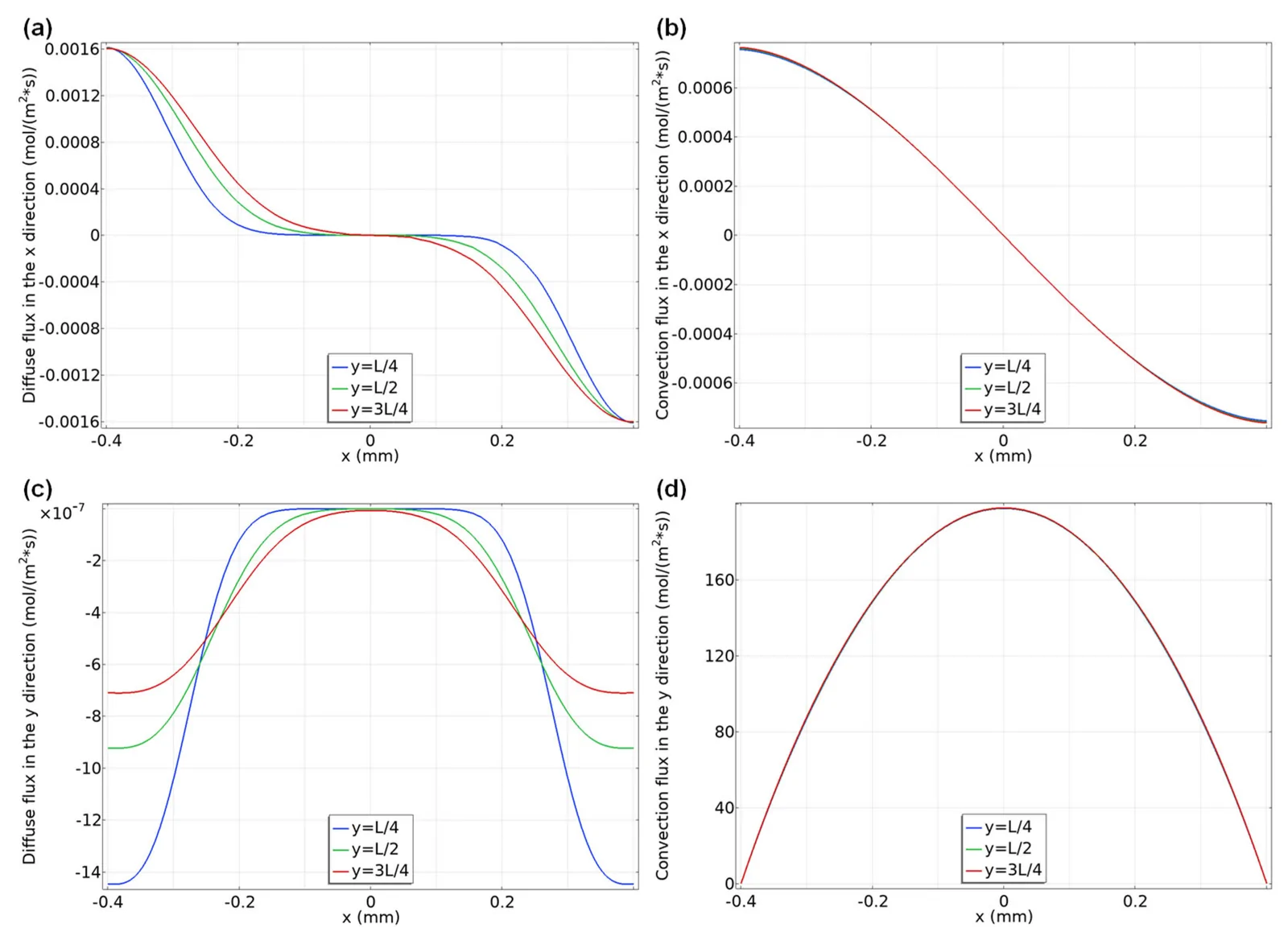
(**a**) Transverse diffusive flux, (**b**) transverse convective flux, (**c**) axial diffusive flux, and (**d**) axial convective flux at different axial positions along the channel (*i* = 400 A/m^2^, *u* = 2.5 cm/s, and LiCl concentration of 20%).

Figure 5 illustrates the ion diffusive and convective flux distributions at three axial locations along the channel, namely *y* = *L*/4, *L*/2, and 3*L*/4. As shown in Figure 5a, the transverse ion diffusive flux across the channel is equal in magnitude but opposite in direction at the AEM and CEM, resulting in an almost zero net flux at the channel centerline. A similar distribution is observed for transverse convective flux, as shown in Figure 5b. The axial ion diffusive flux shown in Figure 5c is several orders of magnitude smaller than the transverse component and decreases gradually along the flow direction, with a maximum magnitude of approximately 1.4 × 10^−6^ mol/m^2^/s. In contrast, the transverse diffusive flux reaches approximately 1.6 × 10^−3^ mol/m^2^/s. This large disparity confirms that axial diffusion is negligible compared with transverse diffusion under the present operating conditions, thereby validating a key assumption of the simplified model. Similarly, the axial convective flux shown in Figure 5d remains small along most of the channel length but becomes non-negligible in regions close to the membrane surfaces, where velocity gradients and concentration gradients are strongly coupled.

These results further confirm that concentration polarization is mainly governed by transport processes within the thin boundary layers adjacent to the ion-exchange membranes. The much larger transverse diffusive flux compared with the axial diffusive flux indicates that concentration gradients are primarily established across the channel width rather than along the flow direction. Meanwhile, axial convection controls the renewal of the bulk solution and limits the extension of the enriched region into the channel center. Therefore, the coupled effects of electromigration, transverse diffusion, and axial convection determine the final concentration distribution in high-salinity ED operation.

### 3.2. Effect of Different Parameters

The influence of key operating parameters on the concentration distribution within the concentrate channel was systematically investigated, including flow velocity, applied current density, and inlet salt concentration of LiCl solution.

**Figure 6:**
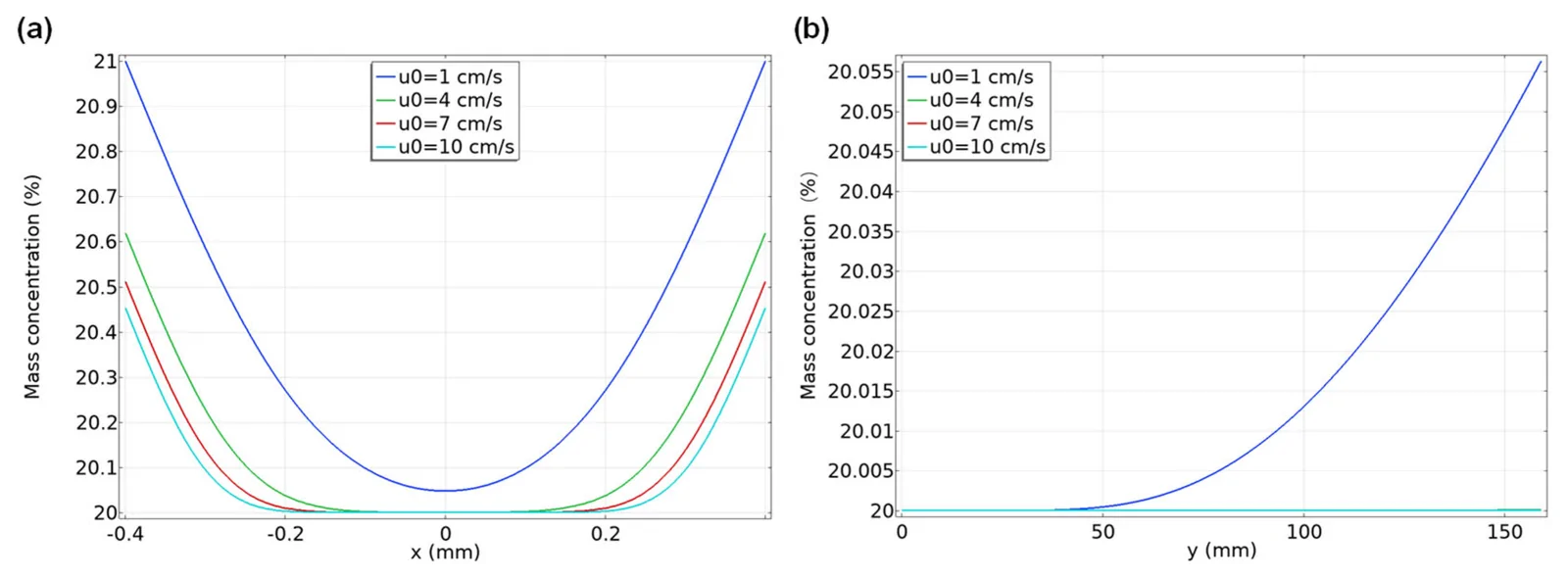
Concentration distribution in the concentrate channel under different flow velocities (*i* = 400 A/m^2^ and LiCl concentration of 20%). (**a**) In the *x* direction (*y* = *L*). (**b**) At the center of the channel in the *y* direction.

Figure 6 illustrates the influence of flow velocity on the concentration distribution within the concentrate channel (simulated with *i* = 400 A/m^2^ and *ξ*_s_ = 20%). As shown in Figure 6a, a lower flow velocity leads to a higher ion concentration near the ion-exchange membrane surfaces at the channel outlet (*y* = *L*), with the surface concentration increasing from approximately 20.45% to 21% as the velocity decreases. In contrast, the outlet concentration at the centerline of the channel remains nearly unchanged for flow velocities between 4 and 10 cm/s. However, when the flow velocity is sufficiently low (e.g., 1 cm/s), corresponding to a longer residence time, the outlet concentration at the channel center can exceed the inlet concentration, as shown in Figure 6b. This occurs because the longer residence time allows more ions, which accumulate near the membrane surfaces due to electromigration, to diffuse from the boundary layer into the bulk flow region. Consequently, the bulk solution becomes more concentrated along the channel length under low-flow conditions.

**Figure 7:**
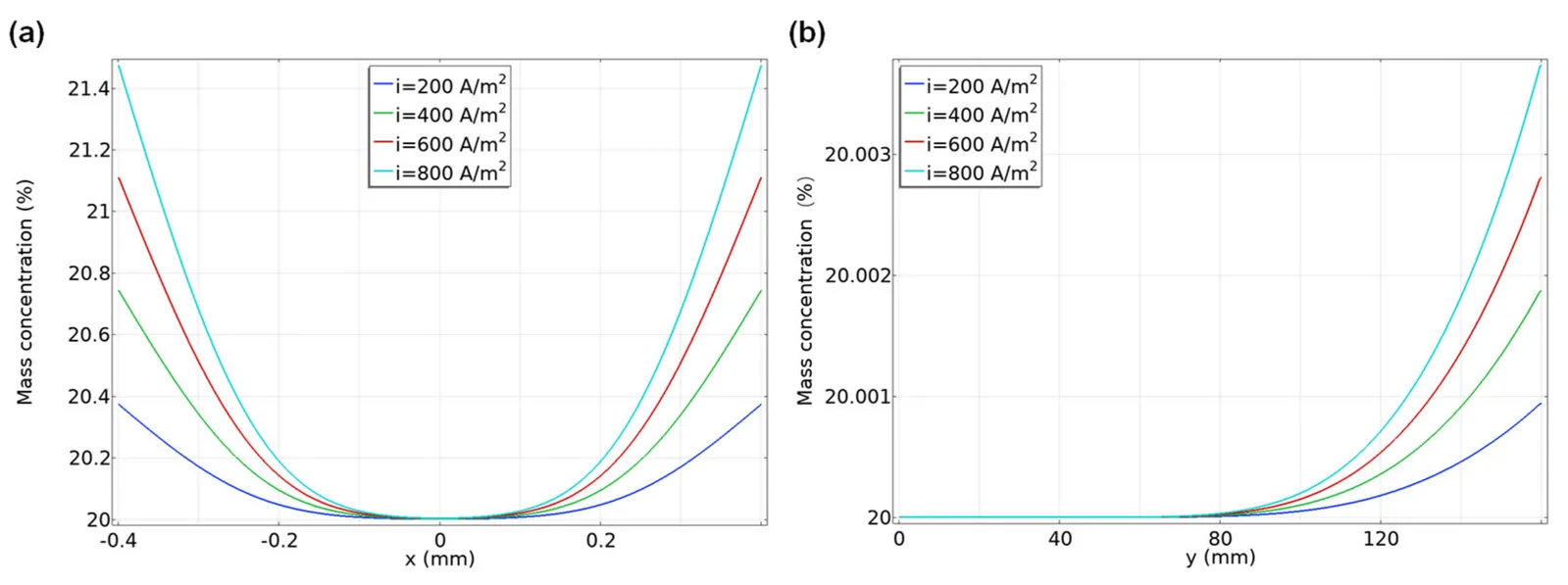
Concentration distribution in the concentrate channel under different current densities (*u* = 2.5 cm/s and LiCl concentration of 20%). (**a**) In the *x* direction (*y* = *L*). (**b**) At the center of the channel in the *y* direction.

Figure 7 illustrates the effect of applied current density on the concentration distribution within the concentrate channel. As shown in the figure, a higher current density results in an increased ion concentration both near the ion-exchange membrane surfaces at the outlet (*y* = *L*), as shown in Figure 7a, and at the centerline of the channel, as shown in Figure 7b. This trend is attributed to the corresponding increase in transmembrane ion migration flux, which is directly proportional to the applied current density. In the context of concentrating high-salinity solutions typically used in liquid desiccant air-conditioning systems, operating at higher current density may therefore be beneficial for achieving faster concentration rates.

However, it is important to note that previous studies have shown that operation at higher current density also leads to increased specific energy consumption [16,37]. Consequently, a practical trade-off must be considered between the desired concentration rate (favored by high current density) and the associated energy consumption. Optimizing this balance is essential for the energy-efficient application of ED in such systems.

**Figure 8:**
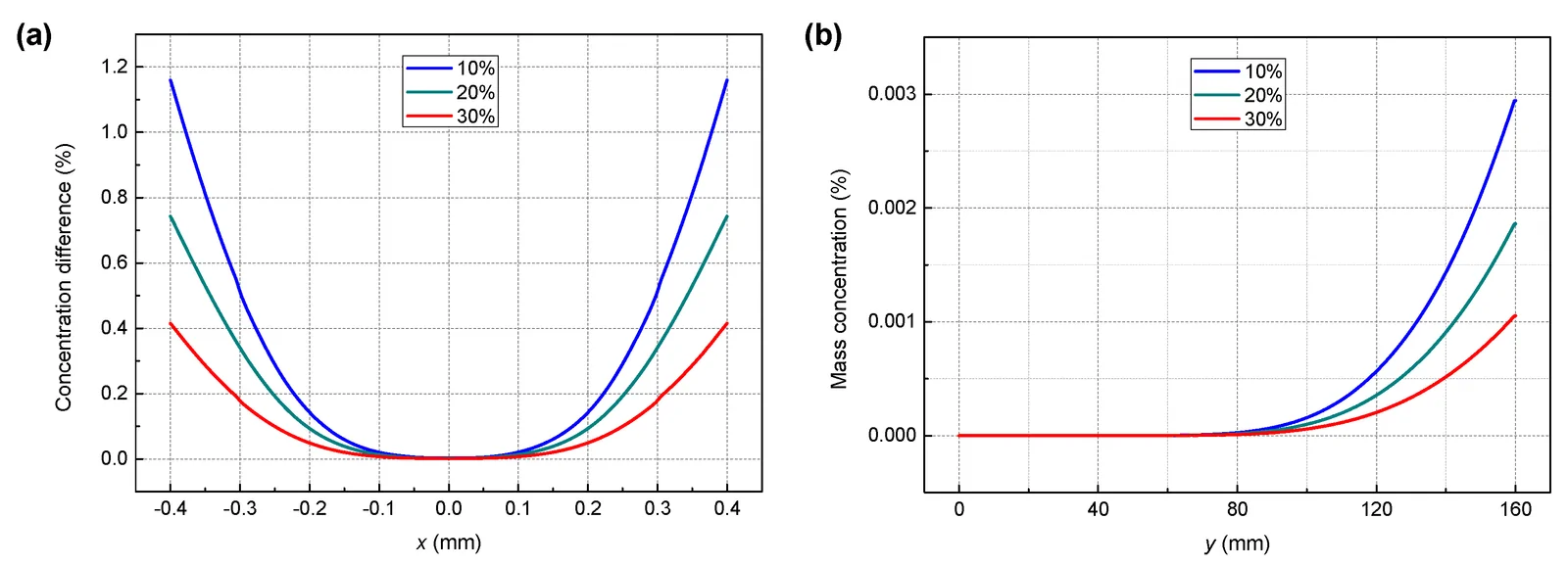
Distribution of the concentration increase from the inlet to the outlet under different inlet LiCl concentrations (*i* = 400 A/m^2^ and u = 2.5 cm/s). (**a**) In the *x* direction (*y* = *L*). (**b**) At the center of the channel in the *y* direction.

Figure 8 illustrates the influence of inlet LiCl concentration on the concentration distribution within the concentrate channel (simulated with *i* = 400 A/m^2^ and *u* = 2.5 cm/s). For clarity, the concentration increase from the inlet to the outlet is calculated and presented for each inlet concentration. As shown in the figure, a higher inlet salt concentration leads to a smaller concentration increase. This trend is observed both near the ion-exchange membrane surfaces at the outlet (*y* = *L*), as shown in Figure 8a, and at the channel centerline, as shown in Figure 8b. The primary reason for this behavior is the dependence of the membrane ion transport number on concentration. The effective salt transport number of the ion-exchange membranes decreases as the bulk salt concentration increases, which in turn reduces the net ion migration flux across the membranes under a given applied current density. Consequently, the degree of concentration achieved per unit channel length decreases at higher inlet concentrations.

At an inlet LiCl concentration of 30%, the model predicts that the concentration near the membrane surface at the outlet is approximately 0.4% higher than the inlet concentration. In the context of conventional ED regeneration for air-conditioning systems, the concentration increase per pass is typically much less than 1% when processing LiCl solutions above 30% [16]. Therefore, achieving a 0.4% concentration increase from a 30% solution in a single pass represents a relatively high-performance level for the ED process. Furthermore, as indicated in the previous section, operating at higher current density can further enhance this concentration increase near the membrane surface. This capability to achieve measurable concentration gains at high salinity is particularly attractive for efficient regeneration of liquid desiccants in air-conditioning applications.

## 4. ED Concentration Using Separate Channels

The preceding analysis demonstrates that, in a conventional ED process, the salt concentration in the bulk flow region remains nearly constant along the flow direction, whereas a significantly elevated concentration develops near the ion-exchange membrane surfaces. This pronounced concentration gradient between the membrane surface and the bulk region inevitably affects the mass transfer performance of the ED process. Furthermore, the concentrated solution within the boundary layer inevitably mixes with the bulk solution at the channel outlet. From a thermodynamic perspective, such mixing introduces an inherent exergy loss, thereby increasing the energy consumption of the separation process.

**Figure 9:**
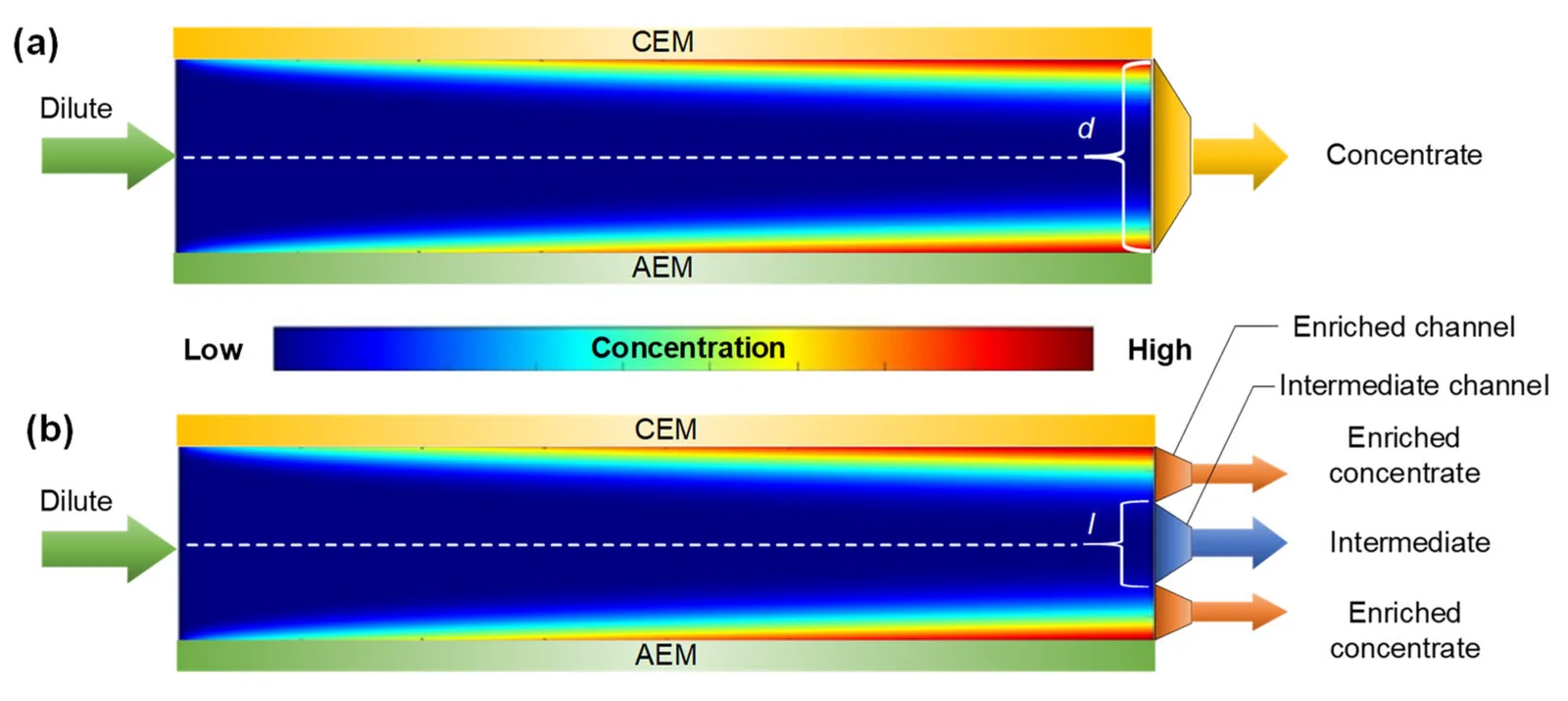
Schematic diagram of (**a**) a conventional ED concentration process and (**b**) an ED concentration process using separate channels.

In recent years, ion concentration polarization (ICP) technologies, which intentionally utilize concentration polarization phenomena, have attracted increasing attention [41,42,43,44,45]. A common strategy employed in these technologies is the use of separate flow channels to independently collect concentrated, diluted, and intermediate streams, thereby minimizing undesired mixing between streams [43]. Figure 9 presents a schematic comparison between a conventional ED concentration process and an ED process employing separate channels.

In the separate-channel configuration shown in Figure 9b, the concentrate flow can be divided into two streams: an enriched concentrate stream near the membrane surface and an intermediate stream in the bulk region. By adjusting the width of the intermediate channel, *l* (*l* ≤ *d*), different combinations of outlet concentration and volumetric flow rate can be achieved for the two streams. This section aims to provide a conceptual analysis of an ED concentration process utilizing separate channels and to explore the potential advantages of actively utilizing concentration polarization for concentrating high-salinity solutions used in air-conditioning systems.

**Figure 10:**
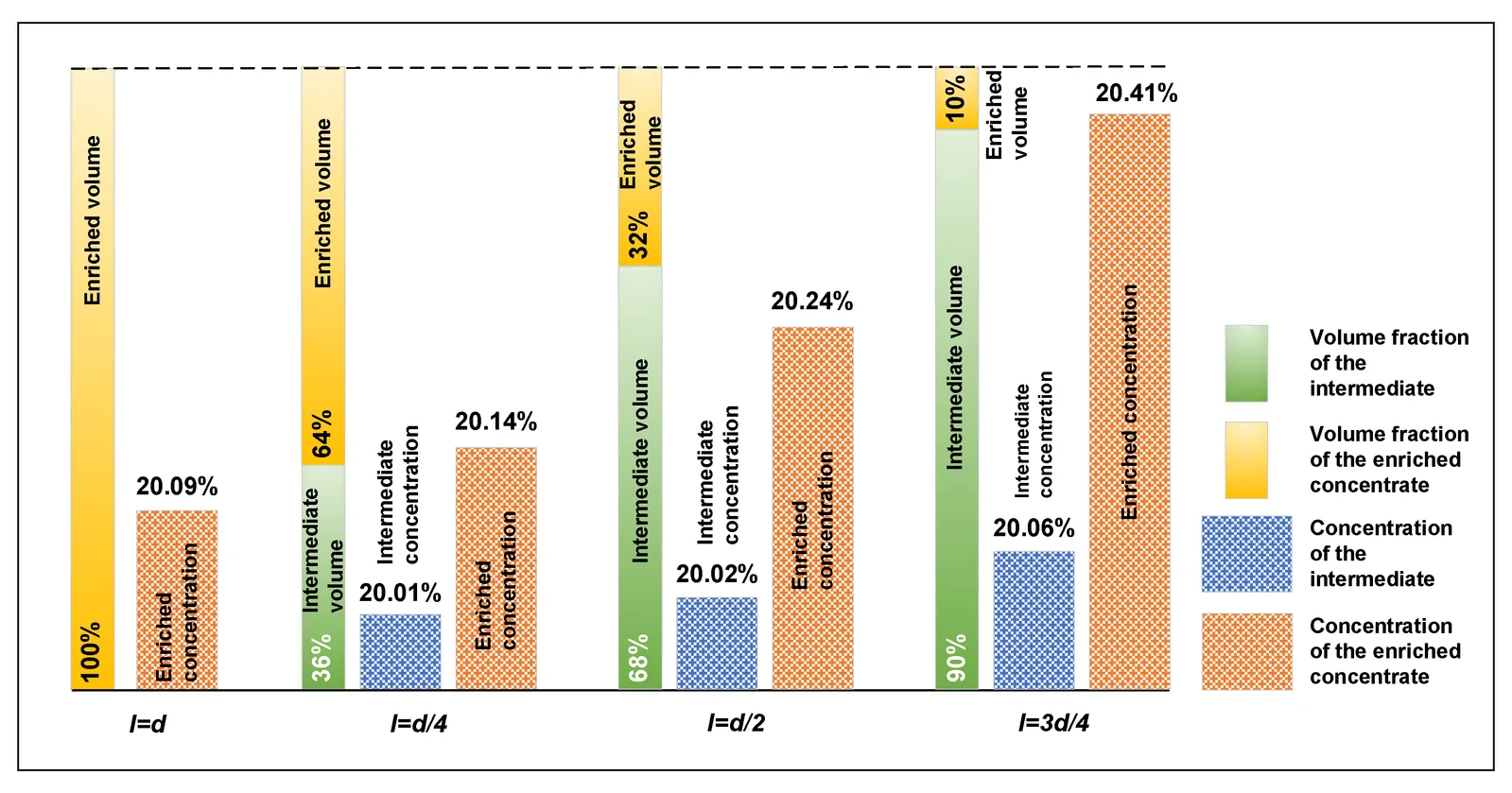
Volume fraction and flow-averaged outlet solute mass fraction of the intermediate and enriched concentrate streams under different intermediate-channel widths. Here, l is the intermediate-channel width and d is the total concentrate-channel width. The intermediate stream represents the bulk-flow region, while the enriched concentrate stream represents the near-membrane boundary-layer region. The dashed line denotes 100% total outlet volume fraction. Operating conditions: inlet LiCl mass fraction $\xi_{s}$ = 20%, inlet velocity u = 2.5 cm/s, and current density i = 400 A/m^2^.

Using Equation (14), the volumetric flow rate and average outlet concentration of each stream can be determined. Figure 10 presents the calculated volume fraction and outlet concentration of both the intermediate stream and the enriched concentrate stream as functions of the relative width of the intermediate channel. For a conventional ED process, where the entire channel width is utilized (*l* = *d*), the average outlet concentration is approximately 20.09%. When the intermediate channel width is set to *l*/*d* = 0.25 the system produces an intermediate stream with a concentration of approximately 20.01% and an enriched concentrate stream with a concentration of approximately 20.14%. The corresponding volume fractions of the intermediate and enriched streams are approximately 36% and 64%, respectively. When *l*/*d* = 0.75 the enriched concentrate stream reaches a concentration of approximately 20.41%, while its volume fraction decreases to only about 10%. These results indicate that the separated-channel design can effectively enhance the concentration level of the enriched stream by mitigating mixing between the boundary-layer and bulk regions. However, this improvement is achieved at the expense of volumetric yield, revealing a clear trade-off between outlet concentration and production capacity.

Similar stream-separation strategies have been experimentally explored in lab-scale ion concentration polarization systems for hypersaline brine treatment. For example, Kim et al. demonstrated a lab-scale ICP device with separate dilute, intermediate, and concentrate channels for treating 70 g/L NaCl brine, suggesting that concentration-polarization-induced stream separation can be physically implemented [43]. Nevertheless, the separated-channel ED configuration proposed in this study remains a preliminary conceptual design. Its practical realization would require further optimization of flow distribution, hydraulic resistance, pressure drop, spacer/support structures, and manufacturability.

Although the separated-channel configuration increases the outlet concentration of the enriched stream, concentration enhancement alone does not necessarily indicate improved overall performance. A complete evaluation should also consider stack voltage, membrane resistance, hydraulic pressure drop, pumping power, product recovery, and specific energy consumption. Therefore, the present analysis should be regarded as a preliminary conceptual assessment, and further experimental and system-level energy analyses are required to evaluate the practical feasibility of this design.

From an application perspective, the separated-channel concept provides a potential way to utilize concentration polarization for enhanced ED concentration performance, but its practical use still requires further structural and hydrodynamic optimization.

## 5. Conclusions

In this study, a simplified model and a coupled model were developed to investigate the concentration and velocity distributions within the concentrate channel of an ED process for high-salinity solution concentration. The comparison between the two models demonstrated that neglecting transmembrane water transport results in an overestimation of ion concentration near the ion-exchange membrane surfaces. In contrast, the coupled model, which incorporates both ion and water transport, provides a more accurate description of concentration polarization behavior within the channel.

The effects of key operating parameters, including flow velocity, applied current density, and inlet salt concentration, on the concentration distribution were systematically analyzed. The results indicate that, under most operating conditions, the concentration in the bulk flow region remains nearly constant along the channel, whereas significantly elevated concentrations develop near the membrane surfaces due to concentration polarization. Lower flow velocity and higher current density enhance ion accumulation near the membrane surfaces, while higher inlet salt concentration reduces the concentration increase achieved within a single pass.

Building on these concentration polarization characteristics, a conceptual ED configuration with separated channels was proposed and analyzed. The results indicate that selectively extracting the enriched boundary-layer region can enhance the concentration of the product stream while mitigating mixing-induced dilution at the outlet. However, this improvement is achieved at the expense of volumetric flow rate, indicating an inherent trade-off between concentration level and production yield.

Overall, this work establishes a modeling framework for predicting concentration distribution in ED channels under high-salinity conditions and provides insight into the role of concentration polarization in ED-based concentration processes in air-conditioning systems. The findings highlight the potential of exploiting, rather than merely limiting, concentration polarization to improve process performance. Future work will focus on optimizing the separated-channel design and experimentally validating the proposed concept under practical operating conditions.

## Data Availability

The raw data supporting the conclusions of this article will be made available by the corresponding author upon request.

## Nomenclature

*C*	concentration, mol/m^3^
*d*	width, m
*D*	diffusion coefficient, m^2^/s
*F*	Faraday constant, C/mol
*i*	current density, A/m^2^
*J*	molar flux, mol/m^2^/s
*l*	length, m
*M*	molar mass, kg/mol
*n*_h_	effective salt hydration number
*p*	pressure, Pa
*t*_s_	salt transport number
*v*	velocity, m/s
*v*_w_	volumetric flux, m^3^/m^2^/s
*V*_m_	molar volume, m^3^/mol
*Greek symbols*	
*ξ*	solute mass concentration
*α*	overall diffusion coefficient, m/s
*β*	overall osmosis coefficient, m^4^/mol/s
*μ*	dynamic viscosity, Pa·s
*ρ*	density, kg/m^3^
*Subscripts*	
ave	average
c	concentrated
d	diluted
s	salt
w	water
*Abbreviations*	
AEM	anion-exchange membrane
CEM	cation-exchange membrane
ED	electrodialysis

## References

[1] Rekhade R., Bose S., Datta S.P., Matuszczak M., Pandelidis D., Jagirdar M. (2025). State-of-the-art on liquid desiccant dehumidification systems: Components, performance, challenges and integration with evaporative cooling systems. J. Build. Eng..

[2] Che C., Qiu K., Wang J., Fan J., Kang Z., Yin Y. (2026). Liquid desiccant materials: A comprehensive review of performance, classification, enhancement, and next-generation design. Energy Build..

[3] Han C., Chen X., Wu S., Ni L. (2024). Experimental investigation on heating performance of a solution regenerator unit using freezing concentration for heat source tower heat pump system. Energy Build..

[4] Huang S., Xie L., Lu L., Zhang X. (2021). Design method for heating towers used in vapor-compression heat pump systems. Build. Environ..

[5] Sun B., Zhang M., Huang S., Su W., Zhou J., Zhang X. (2019). Performance evaluation on regeneration of high-salt solutions used in air conditioning systems by electrodialysis. J. Membr. Sci..

[6] Guan B., Zhang J., Zhang Q., Chang X., Zhang T., Liu X. (2024). Energy and exergy analysis for advanced air-conditioning: Comparative evaluation of electrodialysis and aerodynamic thermal methods in liquid-desiccant reconcentration. Desalination.

[7] Pan X., Liu A., Li J.H., Yang C.L., Liang C.H., Li N.F., Dong Y.F., Dong C.S., Zhao Y.S. (2026). Performance analysis of a solar-driven hollow fiber membrane-based liquid desiccant air-conditioning system in a hot-humid region. Renew. Energy.

[8] Zhai C., Liu B., Xu M., Han H., Sun Y., Wu W. (2025). Liquid desiccant thermal storage driven by off-peak electricity: Synergistic regulation of solution concentration and air humidity for building load shifting. Energy Build..

[9] Cai D., Zhang G., Hu D., Li J., Wang M., Zhang Y., Yuan J. (2023). Efficiently Removing Heavy Metals from High-Salinity Wastewater via Ionic Liquid-Based Aqueous Biphasic Systems. ACS Omega.

[10] Guo L., Xie Y., Sun W., Xu Y., Sun Y. (2023). Research Progress of High-Salinity Wastewater Treatment Technology. Water.

[11] Shi J., Huang W., Han H., Xu C. (2020). Review on treatment technology of salt wastewater in coal chemical industry of China. Desalination.

[12] Liu J., Yuan J., Ji Z., Wang B., Hao Y., Guo X. (2016). Concentrating brine from seawater desalination process by nanofiltration–electrodialysis integrated membrane technology. Desalination.

[13] Inzillo B.M., Aquino M., Quilaqueo M., Straface S., Díaz-Quezada S., Estay H., Argurio P., Santoro S., Curcio E. (2025). Thermo-osmotic membrane distillation-crystallization for enhanced dehydration of lithium brines and sustainable SWRO brine management. J. Water Process Eng..

[14] Guan B., Zhang Q., Li M., Zhang T., Liu X., Wang X. (2024). Investigation on liquid desiccant regeneration for advanced air-conditioning: Aerodynamic thermal method versus mechanical vapor recompression method. Energy Convers. Manag..

[15] Gerges H.A., Attia A., Teamah M.A., El-Maghlany W.M. (2024). Developing an integrated simulator and optimizer model for liquid desiccant dehumidification system “LDDSim”. Therm. Sci. Eng. Prog..

[16] Sun B., Zhang M., Huang S., Wang J., Zhang X. (2021). Limiting concentration during batch electrodialysis process for concentrating high salinity solutions: A theoretical and experimental study. Desalination.

[17] Kim B., Kwak R., Kwon H.J., Pham V.S., Kim M., Al-Anzi B., Lim G., Han J. (2016). Purification of High Salinity Brine by Multi-Stage Ion Concentration Polarization Desalination. Sci. Rep..

[18] Myint M.T., Ghassemi A., Nirmalakhandan N. (2013). Complete sustainability in electrodialysis reversal desalination: Reusing tertiary-treated municipal wastewater as feed in the concentrate stream and electrodes rinsing water. Desalin. Water Treat..

[19] Melnikov S., Mugtamov O., Zabolotsky V. (2020). Study of electrodialysis concentration process of inorganic acids and salts for the two-stage conversion of salts into acids utilizing bipolar electrodialysis. Sep. Purif. Technol..

[20] Sun B., Huang S., Su W., Lu L., Zhang X. (2023). A comprehensive analysis of the minimum energy and thermodynamic efficiency of regenerating aqueous electrolyte solutions in air-conditioning systems. Energy.

[21] Tong T., Elimelech M. (2016). The Global Rise of Zero Liquid Discharge for Wastewater Management: Drivers, Technologies, and Future Directions. Environ. Sci. Technol..

[22] Yan H., Wang Y., Wu L., Shehzad M.A., Jiang C., Fu R., Liu Z., Xu T. (2019). Multistage-batch electrodialysis to concentrate high-salinity solutions: Process optimisation, water transport, and energy consumption. J. Membr. Sci..

[23] Liu L., Cheng Q. (2020). Mass transfer characteristic research on electrodialysis for desalination and regeneration of solution: A comprehensive review. Renew. Sustain. Energy Rev..

[24] Li X.W., Zhang X.S. (2009). Photovoltaic–electrodialysis regeneration method for liquid desiccant cooling system. Sol. Energy.

[25] Guo Y., Al-Jubainawi A., Ma Z. (2019). Performance investigation and optimisation of electrodialysis regeneration for LiCl liquid desiccant cooling systems. Appl. Therm. Eng..

[26] Strathmann H. (2010). Electrodialysis, a mature technology with a multitude of new applications. Desalination.

[27] La Cerva M., Gurreri L., Tedesco M., Cipollina A., Ciofalo M., Tamburini A., Micale G. (2018). Determination of limiting current density and current efficiency in electrodialysis units. Desalination.

[28] Feng X., Huang R.Y. (1994). Concentration polarization in pervaporation separation processes. J. Membr. Sci..

[29] Tanaka Y. (2003). Concentration polarization in ion-exchange membrane electrodialysis—The events arising in a flowing solution in a desalting cell. J. Membr. Sci..

[30] Lokare O.R., Ji P., Wadekar S., Dutt G., Vidic R.D. (2019). Concentration polarization in membrane distillation: I. Development of a laser-based spectrophotometric method for in-situ characterization. J. Membr. Sci..

[31] Sitaraman H., Battiato I. (2022). Impact of Large-Scale Effects on Mass Transfer and Concentration Polarization in Reverse Osmosis Membrane Systems. Sep. Purif. Technol..

[32] Mitko K., Turek M. (2014). Concentration distribution along the electrodialyzer. Desalination.

[33] Law M., Wen T., Solt G. (1997). Thickness and concentration profile of the boundary layer in electrodialysis. Desalination.

[34] Mas L., Pierrard P., Prax P., Sohm J. (1970). Behaviour of an electrodialysis unit cell. Desalination.

[35] Tedesco M., Hamelers H., Biesheuvel P. (2016). Nernst-Planck transport theory for (reverse) electrodialysis: I. Effect of co-ion transport through the membranes. J. Membr. Sci..

[36] Zourmand Z., Faridirad F., Kasiri N., Mohammadi T. (2015). Mass transfer modeling of desalination through an electrodialysis cell. Desalination.

[37] Sun B., Zhang M., Huang S., Cao Z., Lu L., Zhang X. (2022). Study on mass transfer performance and membrane resistance in concentration of high salinity solutions by electrodialysis. Sep. Purif. Technol..

[38] Conde M.R. (2004). Properties of aqueous solutions of lithium and calcium chlorides: Formulations for use in air conditioning equipment design. Int. J. Therm. Sci..

[39] Stokes R.H. (1950). The Diffusion Coefficients of Eight Uni-univalent Electrolytes in Aqueous Solution at 25°. J. Am. Chem. Soc..

[40] Fadaei F., Shirazian S., Ashrafizadeh S.N. (2011). Mass transfer modeling of ion transport through nanoporous media. Desalination.

[41] Kim S.J., Ko S.H., Kang K.H., Han J. (2010). Direct seawater desalination by ion concentration polarization. Nat. Nanotechnol..

[42] Yoon J., Do V.Q., Pham V.S., Han J. (2019). Return flow ion concentration polarization desalination: A new way to enhance electromembrane desalination. Water Res..

[43] Kim B., Kwon H., Ko S.H., Lim G., Han J. (2017). Partial desalination of hypersaline brine by lab-scale ion concentration polarization device. Desalination.

[44] Zhu C., Zuo X., Xian W., Guo Q., Meng Q.W., Wang S., Ma S., Sun Q. (2022). Integration of Thermoelectric Conversion with Reverse Electrodialysis for Mitigating Ion Concentration Polarization and Achieving Enhanced Output Power Density. ACS Energy Lett..

[45] Khatibi M., Aminnia A., Ashrafizadeh S.N. (2024). The role of ionic concentration polarization on the behavior of nanofluidic membranes. Chem. Eng. Process. Process Intensif..

